# Hybrid Label-Free Molecular Microscopies for Simultaneous Visualization of Changes in Cell Wall Polysaccharides of Peach at Single- and Multiple-Cell Levels during Postharvest Storage

**DOI:** 10.3390/cells9030761

**Published:** 2020-03-20

**Authors:** Weinan Huang, Yating Nie, Nan Zhu, Yifan Yang, Changqing Zhu, Minbiao Ji, Di Wu, Kunsong Chen

**Affiliations:** 1 College of Agriculture & Biotechnology/Zhejiang Provincial Key Laboratory of Horticultural Plant Integrative Biology/The State Agriculture Ministry Laboratory of Horticultural Plant Growth, Development and Quality Improvement, Zhejiang University, Zijingang Campus, Hangzhou 310058, China; 11816084@zju.edu.cn (W.H.); 21516049@zju.edu.cn (Y.N.); nnz916@zju.edu.cn (N.Z.); zcq1236@zju.edu.cn (C.Z.); akun@zju.edu.cn (K.C.); 2 State Key Laboratory of Surface Physics and Department of Physics, Human Phenome Institute, Multiscale Research Institute of Complex Systems, Key Laboratory of Micro and Nano Photonic Structures (Ministry of Education), Fudan University, Shanghai 200433, China; 18110190062@fudan.edu.cn (Y.Y.); Minbiaoj@fudan.edu.cn (M.J.)

**Keywords:** peach, softening, cell wall, polysaccharides, confocal Raman microspectroscopy, Fourier transform infrared microspectroscopy, stimulated Raman scattering microscopy

## Abstract

Softening of fruit during the postharvest storage, which is mainly associated with both compositional and spatial changes of polysaccharides within cell wall, affects the texture and quality of fruit. Current research on the fruit softening mechanism lacks an understanding of the overall softening at the cell level. The objective of this work was to investigate the change in the spatial distribution of cell wall polysaccharides in peach flesh cells at both single- and multiple-cell levels in a label-free way during the postharvest storage. Nonmelting peaches (*Prunus persica* L. Batsch cv.”Zhonghuashoutao”) at commercial maturity were stored at 0 °C and 20 °C. Firmness measurement and chemical analysis were performed at each storage time. In addition, three molecular imaging techniques, namely confocal Raman microspectroscopy (CRM), Fourier transform infrared microspectroscopy (FTIRM), and stimulated Raman scattering microscopy (SRS) were used to visualize changes in the spatial distribution of cell wall polysaccharides of peach fruit in a label-free way during the postharvest storage. The combination of CRM and FTIRM provided complementary spectral information to visualize the spatial changes of cellulose, hemicellulose, and pectin in the cell wall of peach flesh during softening at the single-cell level, and found that the cell wall polysaccharides tended to be concentrated in the cell corner of parenchymal cells at the late stage. Furthermore, SRS, which is an ultrafast Raman imaging technique (approximately three or four orders of magnitude faster than CRM), was used for high-throughput cell wall phenotypes measurement. Different degradation degrees of parenchymal cells during fruit softening were found based on the gray-scale statistical analysis of SRS data. In general, cell wall polysaccharides decreased during softening and tended to be concentrated in the cell corner for most parenchymal cells at the late stage, but there were also some cells not in line with the whole softening trends. The results show that there were differences in the content and spatial changes of cell wall polysaccharides among parenchymal cells of peach fruit during the softening process, and the hybrid use of CRM, FTIRM, and SRS is a promising method for simultaneous visualization of changes in cell wall polysaccharides of peach.

## 1. Introduction

Peach, as one of the most favored fruits among consumers worldwide, is rich in nutrition and good flavor. Like most other fruits and vegetables, peach fruit softens during the ripening process [1]. The softening of peach fruit affects its texture, and then significantly influences consumer preference and acceptability [2]. According to previous studies, the textural changes of fruit are closely related to the cell wall polysaccharides [3,4]. The structure and composition of the cell wall determines the mechanical properties of the fruit flesh, and further affects fruit texture. During the development and postharvest of a peach, polysaccharides change substantially, and this process is mainly related to structural and compositional changes of cellulose, hemicellulose, and pectin, i.e., as well as their spatial orientation [5].

To understand the process and mechanism of fruit softening, different chemical methods and microscopic techniques have been applied to investigate changes in the cell wall composition and structure of fruit. However, the extraction process of cell wall compositions by chemical analysis is miscellaneous and inevitably destructive, leading to significant loss of structural information [6]. Other methods, such as fluorescence microscopy and confocal laser scanning microscopy are also commonly used in microstructure studies [7,8]. However, additional staining or labeling procedures are needed when using these techniques to identify specific chemicals. As a result, sample preparation and cell imaging of these techniques can be invasive, time-consuming, costly, and laborious [9]. 

Label-free imaging technologies have an obvious and intuitive advantage that can obtain chemical information of targeted components in samples without additional dye or markers invading and modifying a sample. Confocal Raman microspectroscopy (CRM) and Fourier transform infrared microspectroscopy (FTIRM) are two typical label-free hyperspectral imaging techniques, which are based on collecting Raman spectroscopy (RS) and infrared spectroscopy (IR) information of a sample at the microcosmic level, respectively. RS comes from the inelastic scattering of photons, and IR is based on the excitation of the molecular vibrations generated from the absorbed light of chemical bonds [10]. The combination of RS/IR and microscopy provides more detailed and robust information to characterize samples at the micro scale. CRM has been applied to the observation of fruit samples, such as carotenoids in tomato [11], polysaccharide changes in the cell wall of apple [9], lignification of loquat fruit [12], and chemical compositions in the cell wall of pear infected by fungi [13]. FTIRM has been used to study plants such as Arabidopsis petals [14], crops [15], and olive fruit [16], but not for the postharvest storage of fruit research. In samples, CRM and FTIRM both have the ability to extract the spectral distribution of multiple chemical groups in one measurement.

In addition to imaging cells at the single-cell level using CRM and FTIRM, the imaging of plant tissues must also be performed on a large-scale to study the variations of cells as a whole, to avoid the problem of limited sample representation due to small-scale cell imaging. Stimulated Raman scattering microscopy (SRS) is a state-of-the-art Raman microspectroscopy technique that stimulates the Raman transition of molecules by means of nonlinear interactions with two coherent pulse laser beams [17]. SRS can realize Raman hyperspectral imaging of biomolecules in a large field of view for massive cells in a label-free molecular imaging way, much faster than CRM, while maintaining the same resolution as CRM [18]. Currently, SRS has been mainly used to study animal and human cells. There are only a few works using SRS to study plant cells, let alone fruit.

The analysis of cell wall degradation in the spatial dimension during the postharvest softening process can help to reveal the softening mechanism of fruit. In this work, a nonmelting peach named ”Zhonghuashoutao” was considered. To obtain the texture change of the nonmelting peach fruit during the postharvest storage, their firmness and cell wall polysaccharide content of homogenized bulk tissues were measured. Then, the hybrid label-free hyperspectral molecular imaging techniques of CRM, FTIRM, and SRS were used to present the distribution changes of cell wall compositions of fruit flesh at both the single-cell level and the massive-cell level. To the best of our knowledge, the change in the spatial distribution of cell wall polysaccharides in peach flesh cells during the postharvest storage has not been reported.

## 2. Materials and Methods

### 2.1. Sample Preparation

Nonmelting peaches (*Prunus persica* L. Batsch cv. ”Zhonghuashoutao”) were harvested from an orchard in Laixi, Shandong, China. The fruit was transported to the laboratory on the day of harvest. Fruit of uniform commercial maturity and absence of disease and mechanical wounding was selected, randomly divided into two groups, and stored at 0 °C and 20 °C (85% to 90% RH), respectively. Each group had 120 peach fruit. Because the nonmelting peaches usually have a longer storage life than the melting peaches, the storage time for the nonmelting peaches was set as 60 days (0 °C) and 30 days (20 °C). The fruit in group one was stored at 0 °C and sampled on the 0, 10, 20, 30, 40, 50, and 60 d; the fruit in group two was stored at 20 °C and sampled on the 0, 5, 10, 15, 20, 25, and 30 d. 

For firmness measurements, there were three fruits per replicate and three replicates for each storage time at each temperature, resulting in 63 fruits used for each group (3 fruits × 3 replicates × 7 days). For chemical analysis, the flesh of the same fruit for the firmness measurement, excluding the parts that were penetrated in the measurement, were cut into small cubes, frozen in liquid nitrogen, and stored at –80 °C.

For CRM imaging, the flesh tissue in the equatorial direction was cut into slices of a thickness of 120 μm using a vibratome (LEICA VT 1000 S). The slices were placed on a microscope slide covered with aluminum foil to avoid interference from the glass Raman bands. After sectioning, the slices were dried on air for further Raman imaging. Three images were acquired for each storage time at each temperature.

For the FTIRM imaging, approximately 1 cm^3^ fruit flesh was taken from 0.3 cm below the equatorial surface of peach fruit and placed in an FAA tissue fixative solution for preservation. Then, fruit flesh with wax was sectioned into 8 μm thick slices on gold plated slides. After that, the slides were placed in 100% dimethyl benzene-ethanol solution three times for 5 min each time to remove paraffin wax, followed by 5 min in 100%, 85%, 75%, and 50% ethanol and water solution three times each to remove dimethyl benzene. Finally, ethanol in the slices was removed using distilled water, and stored with silica gel particles after freeze-drying for further FTIRM imaging. 

The slice preparation for SRS analysis was similar to that for Raman imaging. The difference was that for SRS it was necessary to seal the prepared sections between the two cover slides instead of air-drying under natural conditions for CRM. Moreover, to avoid drying the slices caused by laser irradiation, a drop of deionized water was placed on the flesh slice and the slice was sealed between the two cover slides using nail polish. Three FTIRM images and five SRS images were acquired for storage times of 0, 30, and 60 d at 0 °C and for storage times of 0, 15, and 30 d at 20 °C.

### 2.2. Measurement of Firmness and Cell Wall Materials

A TA-XT plus Texture Analyzer (Stable Micro Systems, UK) fitted with a 7.5 mm diameter probe was used to measure the fruit firmness. After removing a slice of peel, firmness was measured on each fruit at each storage time, at three positions that were 120 degrees apart at the fruit equator. The penetration rate was 5 mm s^−1^, the penetration depth was 5 mm, and the data were expressed in newtons (N). 

The modified hot alcohol-insoluble solids method was used in the experiment, which was proposed by Renard [19]. The cell wall material (CWM) was obtained from the peeled and nucleated parenchyma tissue for use in the analysis of their chemical composition. The determination of cell wall fractions was carried out on the basis of the method described in Figueroa et al. [20] and Vicente et al. [21]. In brief, the supernatants of water soluble pectins (WSP), chelator soluble pectins (CSP), diluted alkali soluble pectins (DASP), and hemicellulose were acquired after sequential extraction from the CWM by deionized water, chelating agent, and dilute alkali. The pectin content in the CWM of the sample was reflected by the content of galacturonic acid (GalA), and the GalA content in the WSP, CSP, and DASP fractions was performed with the carbazole method [22]. Because xyloglucan is the main hemicellulose in primary cell walls of dicots [23,24,25] and peach belongs to the class dicotyledon, meanwhile cells present in fruit flesh mainly have primary cell wall [26,27], for the hemicellulose measurement, xyloglucan was regarded as the main hemicellulose in the cell wall of nonmelting peach. Therefore, the hemicellulose content was reflected by the xyloglucan content. Although xyloglucan involves other sugars (xylose, galactose), as xyloglucan is composed of a backbone of glucose [28,29,30], the content of xyloglucan was determined by measuring the glucose content using the anthrone method [20]. D-(+) GalA and glucose were used as the standard, respectively. The colorimetric determination of GalA and glucose was conducted with a BioTek synergy4 multimode microplate reader (BioTek, USA) at 525 and 620 nm, respectively. The cellulose content was determined by the gravimetric method. The contents of cellulose, hemicellulose, and pectin in the sample were expressed as mg per g CWM dry weight.

### 2.3. Label-Free Hyperspectral Molecular Imaging

The methods of CRM hyperspectral imaging and the reference RS of the cellulose and pectin were performed according to our previous work [12]. A Renishaw inVia Reflex Raman Microscopy System (Renishaw Plc., Wotton-Under-Edge, UK) equipped with a 532 nm diode laser and an air-cooled charge-coupled device was used. The spectrometer was equipped with two gratings (1200 mm/line (visible) and 2400 mm/line (NIR)). Leica DMLM fitted with four objectives (×100/0.75 NA, ×50/0.75 NA, ×20/0.40 NA, and ×5/0.12 NA) comprised the attached microscope. The images were recorded at a spatial resolution of 2 μm in both directions, the x and y, and the z direction were fixed. The spectra were collected over the spectral range from 1800 to 600 cm^−1^ under the 50× objective in static mode. In most previous works of CRM imaging of cell wall, the exposure time was usually set from 3 to 10 s and the laser power was set from 10 to 50 mW. However, because the parenchyma cells of fruits have thinner cell walls than other kinds of plant cells, a low exposure time would cause the RS signal of cell wall material of parenchyma cells to be too weak to be detected. Therefore, different exposure times (0.01 to 10 s) and laser power (1 to 50 mW) were evaluated, and the optimal exposure time and laser power for CRM acquisition of parenchyma cell walls of peach were determined to be 6 s and 50 mW, respectively.

The FTIRM hyperspectral imaging system used in this experiment was Nicolet iN10 (Thermo Fisher Scientific., USA). A mercury cadmium telluride was set up to conduct imaging and was cooled by liquid nitrogen before measurement. IR acquisition and processing (automatic balance, automatic smoothing, etc.) were all performed using an OMNIC Picta™ (Thermo Fisher Scientific, Waltham, MA, USA). The sample was observed on the loading platform with a charge-coupled device camera, and the area of interest was selected for spectral analyses. Spectral data were collected using reflection mode, and the spectrum was measured in the spectral range of 4000–600 cm^−1^ with a spectral resolution of 8 cm^−1^. Spectra were collected from each point at an aperture of 10 × 10 μm with a 10 μm scanning step in both the x-axis and y-axis directions; 256 scans per spectrum were accumulated. FTIRM hyperspectral imaging was constructed according to the intensity of the selected wavenumber.

The construction of the SRS images was achieved by two-dimensional galvanometer repetitive scanning of a large number of pump and Stokes pulses. A microscope objective focused the peach sample when the pump and Stokes pulse would temporally and spatially coincident. High-repetition-frequency intensity modulation was carried out on the Stokes or pump light, and then the other light was applied to the lock-in amplifier to measure the spectrum. For SRS imaging, the central wavelength of the pump light was adjusted to 802 nm, which made the difference between the frequencies of the Stokes light wavelength close to the Raman characteristic peak of the cell wall polysaccharide composition at 2900 cm^–1^. All SRS images were obtained under the parametric conditions of the pump light center wavelength at 802 nm and the stage position of 25. The 2900 cm^–1^ peak was chosen for the acquisition of SRS images. Each large-scale image obtained by SRS was composed of 512 × 512 pixels with a size of 423.94 × 423.94 μm. The time for spectral acquisition at each pixel was approximately 50 μs. A total of 7 × 7 SRS images were acquired at each measurement with three replicates for each postharvest stage under two temperatures. Then, the acquired images and the spectra of the images were joined, and then extracted with ImageJ (National Institute of Mental Health, USA).

### 2.4. Statistical Analysis

SPSS 16.0 (SPSS, Chicago, IL, USA) was used for the descriptive statistical analysis, the analysis of the variance (one-way ANOVA) and the post hoc Tukey’s honestly significant difference test (HSD). Origin 9.0 (Microcal Software Inc., Northhampton, MA, USA) was used to prepare the figures.

## 3. Results

### 3.1. Firmness and Cell Wall Polysaccharide Content

**Figure 1:**
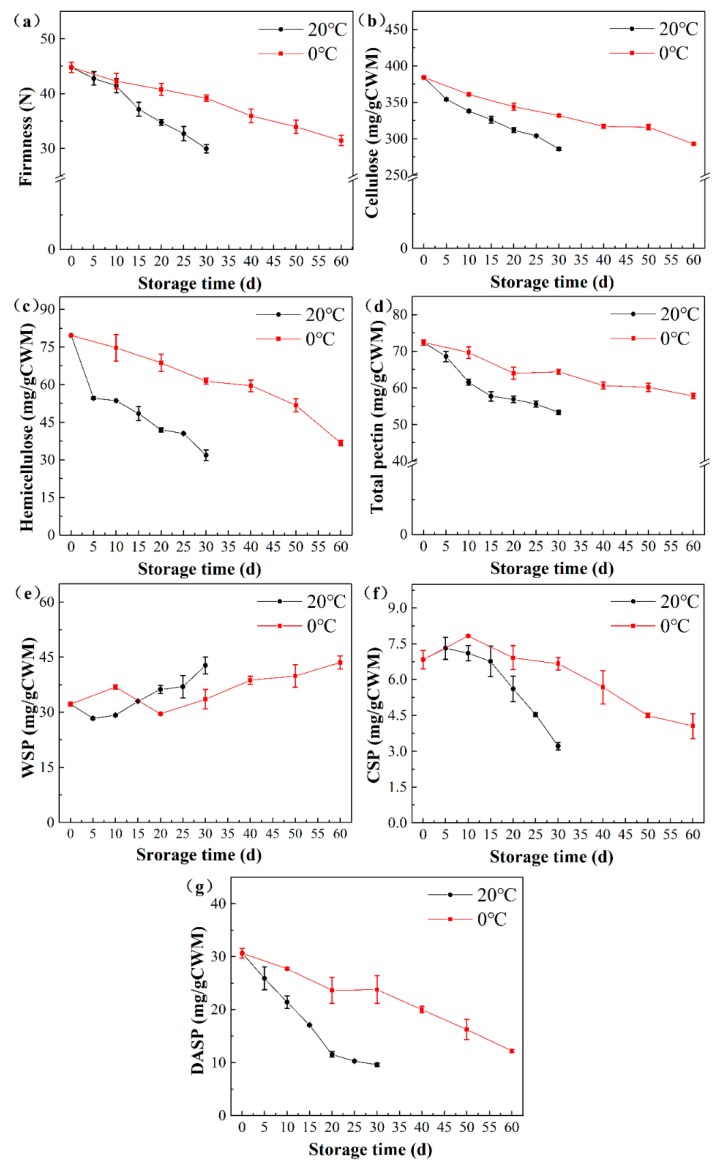
Changes in firmness (**a**) and content of cellulose (**b**), hemicellulose (**c**), total pectin (**d**), and three fractions of pectins (WSP (**e**), CSP (**f**), DASP (**g**)) for the peach of ”Zhonghuashoutao” cultivar stored at 0 °C and 20 °C. The hemicellulose content was reflected by the xyloglucan content, which was determined by measuring the glucose content using the anthrone method. The bars represent standard.

The firmness of peach fruit stored at 0 °C and 20 °C showed a similar trend of decrease (Figure 1a). The decrease rate was slower at first, and then faster. The firmness was 45.92 N (±2.79) on the first day of storage. The firmness at 0 °C and 20 °C was 65.58% and 71.41% that of the original levels after 60 d and 30 d of storage, respectively.

In general, the cellulose content showed a tendency to decrease at a constant rate (Figure 1b). The content of cellulose in peach stored at 20 °C decreased by 25.52%, from 384.74 to 286.56 mg/g CWM, during the whole storage period of 30 d. The content of cellulose in peach stored at 0 °C decreased slower than that of 20 °C. On the 30th d, the decrease in cellulose content in peach stored at 0 °C was only 51.92% of that of fruit stored at 20 °C. As shown in Figure 1c, the decrease rate of hemicellulose content in peach stored at 20 °C was fast in the first 5 d followed by a slower downward trend between the 5th d and 30th d. In contrast, the hemicellulose content in peach stored at 0 °C decreased steadily. The content of hemicellulose in peach stored at 0 °C on the 60th d (36.73 mg/g CWM) was similar to that stored at 20 °C on the 30th d (31.87 mg/g CWM). 

The content of total pectin and three fractions (WSP, CSP, and DASP) are also shown in Figure 1d–g. The decreasing trends of the total pectin in peach at the two temperatures during storage were similar to those of firmness, cellulose, and hemicellulose content. The WSP fraction content of peach fruit stored at 0 and 20 °C showed a general trend of increase after 5 d and 20 d, respectively. The CSP fraction content increased slightly during the first 5 d and 10 d during storage at 20 °C and 0 °C, respectively. As the storage time increased, the CSP fraction content decreased rapidly for storage at 20 °C, whereas the fraction stored at 0 °C declined much slower. The DASP fraction content continued to decrease during storage, and the rate of decline during storage at 20 °C was faster than that during storage at 0 °C.

In addition, the nonmelting peach, ”Zhonghuashoutao” did not rot after a storage of 60 d at 0 °C or a storage of 30 d at 20 °C, and no chilling symptoms occurred in peach fruit during the whole storage.

### 3.2. CRM Hyperspectral Imaging

#### 3.2.1. Assignment of Characteristic Peaks in RS

All the original RS data of the parenchymal cell wall of peach flesh were eliminated random noise by Savitzky-Golay [12] (Figure S1a), and deduct the interference of the fluorescence background to the RS analysis signal and achieve baseline correction by adaptive iteratively reweighted penalized least squares [31] (Figure S1b). A typical RS profile of the parenchymal cell wall of peach flesh at 0 d is shown in Figure S2. The most prominent Raman band used to identify pectin was at 852 cm^−1^, which was attributed to the vibration of the α-glycosidic bond in pectin [32]. Another characteristic band for pectins was at 1750 cm^−1^, which was generated from the C=O stretching vibration of the ester carbonyl group [9]. Raman bands at approximately 1098 and 1124 cm^−1^ were usually assigned as the RS characteristic bands of cellulose, with a small influence of the hemicellulose [33]. They were characteristic of asymmetric and symmetric stretching vibrations of C-O-C glycosidic bonds in cellulose, respectively [33]. In addition, the RS characteristic bands of cellulose also included 1383 and 1480 cm^−1^. The 1383 cm^−1^ band came from the HCH and HOC bending, and the 1480 cm^−1^ band was assigned to the HCC, HCO, and HOC bending [12]. The most significant characteristic bands of xyloglucan were assigned as the peaks centered at 757 cm^−1^ [9]. However, the intensity of the 757 cm^−1^ band was not detectable in the RS of the parenchymal cell wall of peach flesh, as shown in Figure S2. Therefore, it was not possible to use the band at 757 cm^−1^ to visualize xyloglucan distribution. It should be noted that no xyloglucan signal at 757 cm^−1^ does not mean that xyloglucan was absent in the cell wall of peach. Although xyloglucan could be assigned as the peak centered at 757 cm^−1^ based on the analysis of reference Raman spectra of commercially available xyloglucan [9], it is common that the RS signal at 757 cm^−1^ about xyloglucan is hard to detect in fruit cell wall. In previous works, RS was used to measure the cell walls of tomato [34] and apple [9], where the xyloglucan signal at 757 cm^−1^ was also not detectable in both works.

#### 3.2.2. CRM Hyperspectral Images of Cell Wall Polysaccharides

**Figure 2:**
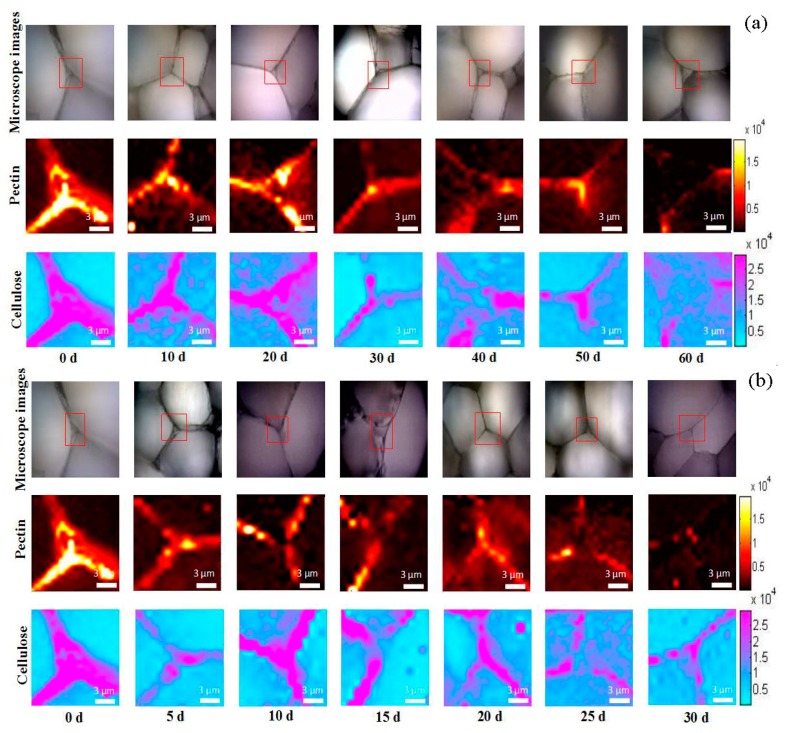
CRM hyperspectral images of cell wall in peach during the postharvest at 0 °C (**a**) and 20 °C (**b**). The Raman images were obtained by integrating Raman bands from 1040 to 1200 cm^−1^ for cellulose, and from 840 to 880 cm^−1^ for pectin. The red squares on the microscope images mark the measurement areas. The red squares on the microscope images mark the measurement areas. Since 0 d is the starting point of the postharvest storage, images at 0 °C and 20 °C on 0 d were the same as they were obtained from the same samples.

CRM was used to visualize the distribution of cellulose and pectin in the cell corner simultaneously according to the Raman characteristic bands of cellulose at 1098 cm^−1^ and 1120 cm^−1^ and pectin at 854 cm^−1^. The molecular Raman images of cellulose and pectin were obtained by integrating over the wavelength ranges centered at these characteristic bands, which were from 1040 to 1175 cm^−1^ for cellulose and from 844 to 864 cm^−1^ for pectin. The distribution of the cellulose and pectin in the cell wall of peach stored at 0 °C are shown in Figure 2a. At the initial stage of storage, the distributions of cellulose and pectin were similar. The high signal intensities of both cellulose and pectin covered the whole cell wall area. In specific, high signal intensity of cellulose over 2 × 10^4^ evenly distributed along the whole cell wall and high signal intensities of pectin (over 1 × 10^4^) also covered the whole cell wall area. Only higher signal intensity of pectin over 1.5 × 10^4^ was concentrated at middle lamella and cell corner. With the prolongation of storage time, the pectin began to degrade. At the late stage of storage, pectin was distributed only at the cell corner. The changes in the distribution of cellulose were similar to that of pectin, i.e., from along the cell wall at the initial storage to only a high signal intensity at the cell corner at the end of storage. 

Figure 2b shows the distribution of the cellulose and pectin in the cell wall of peaches stored at 20 °C. It was found that the changes in the distribution of pectin and cellulose under the storage condition of 20 °C had a similar decline trend to those at 0 °C. The main difference was that the cell wall polysaccharides of peach at 20 °C decreased faster. The degree of degradation of pectin on the cell wall of peach fruit after 60 d of storage at 0 °C and 30 d storage at 20 °C was similar. In addition, at the same storage temperature, the degradation rate of pectin was faster than that of cellulose at 20 °C. Compared with pectin, cellulose also underwent the degradation process, but it still maintained a high Raman signal intensity at the late stage. The change in the content-distribution images provided by CRM had the similar trends of the chemical analysis results.

### 3.3. FTIRM Hyperspectral Imaging 

#### 3.3.1. Analysis of IR of Peach Cell Wall 

Raw IR data of the parenchymal cell wall of the peach flesh were preprocessed by the OMNIC Picta™ to correct baseline and eliminate random noise. Figure S3a shows a typical IR from the peach fruit cell wall. The IR band near 1240 cm^−1^ is the cellulose characteristic peak caused by C-O-C vibration [35]. Pectin has a characteristic peak related to its degree of esterification at approximately 1750 cm^−1^, which was attributed to carboxylic acid and carboxylate groups [36]. Hemicellulose has a complicated absorption pattern in the IR band between 900 cm^−1^ and 1200 cm^−1^ [35]. In addition, pectin also has absorption in this IR range [15]. To further determine the characteristic absorption peak of hemicellulose in peach fruit for imaging, hemicellulose and pectin were extracted from peach flesh and their IR was detected separately. Specifically, the pectin of peach flesh was extracted by water before being coagulated by ethanol [37] and high purity hemicellulose of peach flesh was extracted by alkaline [38]. By comparing the IR profiles of hemicellulose and pectin extracted from peach flesh (Figure S3b), the peak at 1040 cm^−1^ in the IR of hemicellulose was significantly higher than that of pectin. Therefore, the peak at 1040 cm^−1^ was considered the characteristic peak of hemicellulos and the visualization of hemicellulose content based on this peak would not be affected by the influence of pectin. In previous works, IR has also been used to detect hemicellulose signal at 1040 cm^−1^ in other plant materials [35], such as corn [15] and wheat [39]. It should be noted that the assignment of IR peak at 1040 cm^−1^, as hemicellulose relies solely on its appearance specifically in the alkali extractable fraction, is an indication of its hemicellulose identity but not a true confirmation.

#### 3.3.2. FTIRM Hyperspectral Images of Cell Wall Polysaccharides

**Figure 3:**
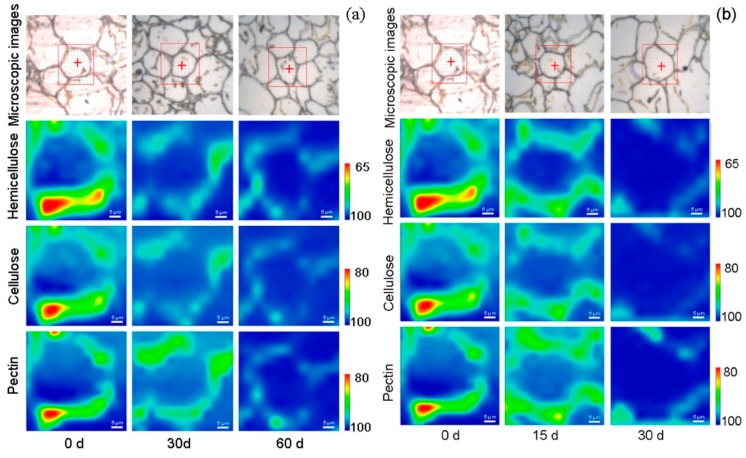
FTIR hyperspectral images of cell wall in peach parenchyma tissue during the postharvest at 0 °C (**a**) and 20 °C (**b**). Spectral images from 1030 cm^−1^ to 1050 cm^−1^, from 1230 cm^−1^ to 1250 cm^−1^, and from 1740 cm^−1^ to 1760 cm^−1^ were showed the distribution of hemicellulose, cellulose and pectin, respectively. The red squares on the microscope images mark the measurement areas. Since 0 d is the starting point of the postharvest storage, images of 0 °C and 20 °C at 0 d were the same as they were obtained from the same samples.

The IR band from 1030 cm^−1^ to 1050 cm^−1^, 1230 cm^−1^ to 1250 cm^−1^, and from 1740 cm^−1^ to 1760 cm^−1^ was used to visualize the distribution of hemicellulose, cellulose, and pectin in the peach cell wall, respectively. Figure 3a,b show FTIRM hyperspectral images of different polysaccharides in the cell wall of peach fruit during 0 and 20 °C storage, respectively. At the initial storage of both temperatures, the main polysaccharide compositions of the cell wall, hemicellulose, pectin, and cellulose were distributed throughout the cell wall and had high infrared signal intensity, especially in the cell corner. During the softening process, the distribution of polysaccharides weakened, which indicates that the degradation of cell wall polysaccharides was accelerated, the signal contour of cell wall polysaccharides distributed along the cell wall was no longer intact, and the degradation rate of pectin was slower than those of hemicellulose and cellulose. At later storage and at both temperatures, hemicellulose, cellulose, and pectin were significantly degraded, and the degradation degree of pectin was slightly lower than those of the other two polysaccharides. However, the IR intensity of these polysaccharides at the cell corner was still higher than other regions on the cell wall. 

In general, the trends of distribution and content of the main cell wall polysaccharides of peach fruit stored at 0 °C and 20 °C were similar to those of CRM results. The main difference between the two temperatures was the rate of cell wall degradation. 

### 3.4. SRS Hyperspectral Imaging

#### 3.4.1. Analysis of SRS Images 

**Figure 4:**
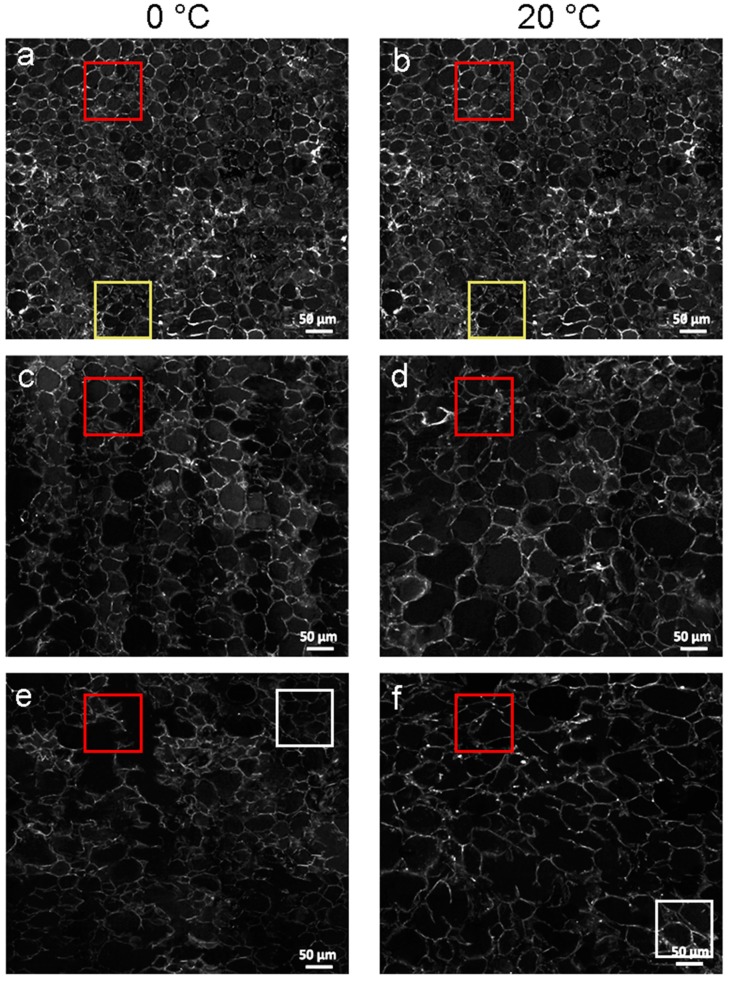
SRS hyperspectral images of peach parenchyma tissue during the postharvest storage at 0 °C and 20 °C. Areas in red box were selected to show the details of the SRS hyperspectral images in Figure S4. Each storage temperature had three stages of data sampling, namely 0, 30, and 60 d for the storage at 0 °C (**a**, **c** and **e**), and 0, 15, and 30 d (**b**, **d** and **f**) for the storage at 20 °C. Areas in yellow box (**a** and **b**) were selected to show cells with relaxing and irregular cell walls at the initial storage. Areas in white box (**e** and **f**) were selected to show cells still having integrate and regular cell walls at the late stage. Since 0 d is the starting point of the postharvest storage, images of 0 °C (**a**) and 20 °C (**b**) at 0 d were the same as they were obtained from the same samples.

The characteristic spectral peak of cell wall polysaccharide compositions with high signal intensity at 2900 cm^−1^ was selected for imaging, which belongs to the CH and CH_2_ stretching of the total polysaccharide composition of cell wall [40,41]. Each SRS image was a combination of a series of views, which contained hundreds of cells from one slice of peach flesh. Large-scale SRS hyperspectral images, as shown in Figure 4, visualized the changes in the distribution and content of cell wall polysaccharides of peach fruit during the softening process at 0 °C and 20 °C, respectively. In general, the cell walls in peach were intact and compact with the high SRS signal in the SRS hyperspectral image at 0 d. With the increase in storage time, the SRS signal of cell wall polysaccharides decreased, and the cell wall relaxed and broke down. The SRS hyperspectral image of peach storage at 0 °C for 60 d was similar to that of storage at 20 °C for 30 d, which suggested that 0 °C inhibited the softening process of peach. The change trend in the cell morphology and SRS signal of cell wall polysaccharides revealed by SRS images was consistent with the results of firmness and cell wall polysaccharides content. Moreover, the SRS images also indicated that the degradation rate of cell walls and their time to start degradation were different (Figure 4). For example, at the initial storage, several cells already had relaxing and irregular cell walls (examples in the yellow box in Figure 4a,b), whereas, some cells still remained integrate and regular cell walls at the late stage (examples in the white box in Figure 4e,f).

In addition to having the capability of rapid imaging the large-scale hyperspectral images can cover hundreds of cells, SRS also possesses the same high resolution as CRM and FTIRM. Figure S4 shows the enlarged views of red box regions in Figure 4. At the initial storage (Figure S4a,b), the cell wall was regular and rigid, and the cell wall polysaccharides were evenly distributed along the cell walls with a high signal intensity. Then (Figure S4c,d), the cell walls begin to relax and expand, and were accompanied by a decrease in the signal intensity of the polysaccharide. Finally, the cell walls began to become irregular, and the signal intensity of polysaccharides signal significantly decreased, except for a high signal intensity still at some cell corner (Figure S4e,f). The individual cell wall information acquired by SRS (Figure S4) agreed with the results provided by CRM and FTIRM (Figure 2 and Figure 3).

#### 3.4.2. Statistical Analysis of SRS Data

In addition to visual observation on SRS images, quantitative and compact representations of cell wall changes during the softening process were carried out by statistical methods. The signal intensity of the cell wall polysaccharide of peach fruit in SRS hyperspectral images was expressed in gray values. Due to the nonuniform distribution of the cell wall polysaccharides of peach fruit, there were various gray values in the same image or even in the same cell. The histogram of each SRS hyperspectral image at each storage time and each temperature is shown in Figure S5. There are differences in the histogram curves for different storage times. In general, the proportion of pixels with low gray values increased as storage time progressed. It can be seen that especially for the pixels with gray values larger than 100, the histogram curves from the SRS image on the 0 d storage are higher than those at the late stage at both 0 °C and 20 °C. 

**Figure 5:**
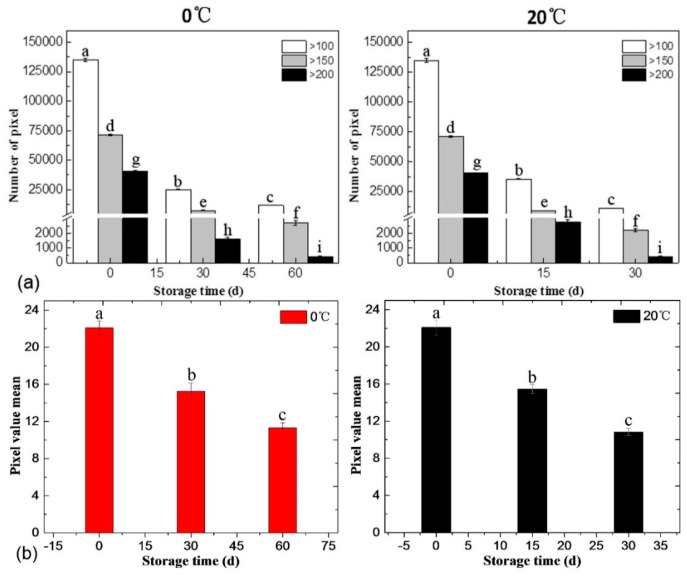
Statistics of pixel value distribution (**a**) and pixel value mean (**b**) in the SRS hyperspectral image of peach stored at 0 °C and 20 °C. The bars represent standard deviation. The same superscript letters above the bars mean no significant difference at *p* = 0.05. The effect is significant with *p* < 0.05.

To further analyze SRS hyperspectral images in order to quantitatively evaluate the softening process of peach flesh, the average gray value of each image was calculated and the numbers of pixels with gray values higher than three thresholds (>100, >150, >200) were also counted. As shown in Figure 5a, at the initial stage of softening, the number of pixels with gray values higher than 100 was as high as 134,960 (± 1419). At the end of storage at 0 °C, the number of pixels with gray values higher than 100 decreased to 11,797 (± 216), which was only approximately 8.74% of that at the initial stage. Similarly, the number of pixels with gray values higher than 100 also decreased to 8.10% during the softening process at 20 °C (10,927 (± 205) pixels at the end of storage vs. 134960 (± 1419) pixels at the initial stage). The numbers of pixels with gray values higher than 150 and 200 in the SRS hyperspectral images of peach fruit stored at 0 °C decreased by 96.21% and 98.92%, respectively, from the initial stage to the late stage. Similarly, for the 20 °C group, the numbers of pixels with gray values higher than 150 and 200 in the SRS images decreased by 95.49% and 98.99%, from the initial stage to the late stage.

Overall, the trend of gray peach values was consistent with the trend of softening peach changes. Both the lower average gray values of cell wall obtained by SRS and cell wall polysaccharides contents in flesh measured by biochemical analysis at the end of storage indicated that the cell wall polysaccharides were degraded during storage as compared with the beginning of storage. The average gray values of peaches stored at both 0 °C and 20 °C, on the one hand, decreased constantly during the storage (Figure 5b), which were similar to the contents of cellulose, hemicellulose, and total pectin (Figure 1b,c,d). The firmness also had similar decreasing trends as compared with average gray values. On the other hand, the decrease rates of these indexes at 0 °C were all about half those of 20 °C. The average gray values of peaches stored at 0 °C and 20 °C dropped by 31.22% and 30.32% in the middle stage and 48.87% and 51.13% at the late stage, respectively (Figure 5b). The decrease percentages of cellulose, hemicellulose, and total pectin content also had the same patterns. At the end of storage, the average gray value of peaches stored at 0 °C on 60 d was similar to that at 20 °C on 30 d, whereas the contents of cellulose, hemicellulose, and total pectin of peaches stored at 0 °C on 60 d were also similar to those at 20 °C on 30 d.

## 4. Discussion

CRM has been used to identify changes in the pear cell wall after infection by fungi [13] and the compositions of lignified cells in loquat fruit [12]. In particular, Szymańska-Chargot, Chylińska, Pieczywek, Rösch, Schmitt, Popp, and Zdunek [9] applied the CRM to image the cellulose and pectin in apples during development and senescence. The study found that at the end of storage, the pectin of apple flesh was mainly dispersed rather than concentrated in the cell wall. However, we found that the pectin of peach flesh remained mainly at the cell corner, showing that the pectin changes in the softening process of apples and peaches are not exactly the same. Due to the similar chemical and structural composition between the cellulose and xyloglucan, their Raman peaks often overlap and are not easily distinguishable. Because RS could not detect the signal of xyloglucan to obtain the distribution of hemicellulose in cell wall of nonmelting peach, FTIRM was used to obtain the distribution of hemicellulose. The use of FTIRM resulted in changes in the compositional, content, and distribution of the cell wall polysaccharides in peach fruit during postharvest storage, which not only were basically consistent with CRM results on cellulose and pectin but also complemented the distribution of hemicellulose that can hardly be identified by CRM. In addition, the measuring environment of FTIRM is not as amenable as CRM for biological samples, because water and carbon dioxide have a great influence on the IR measurement of biological samples. Therefore, the combination of CRM and FTIRM was suggested to study the changes of different polysaccharides in fruit softening.

As shown in Figure 4, the cells of peach flesh were found to have different degrees of degradation during the softening process. The analysis based on a large number of cells is important to provide the capability of observing more general and representative trends of fruit softening. Although the imaging of cell wall polysaccharides at high resolution was performed by CRM and FTIRM, their measured images covered only a small field of view with a few cells. As an advanced Raman-based microscopy technique, SRS offers unprecedented fast imaging and also expands the measured vision fields from a few cells to hundreds of cells while identifying the distribution and content information of specific chemicals in situ. The imaging speed of SRS was approximately three to four orders of magnitude faster than CRMs. SRS-based large-scale hyperspectral imaging enables high-throughput expression of cell phenotypes in images, enabling multidimensional data analysis from multiple angles. This was the first time SRS was applied on studying fruit cells. 

The SRS images of peach cells at different storage times show that the degradation of each cell wall did not all begin when the fruit begins to soften, and the rate of degradation of each cell wall was not consistent with the overall rate of fruit softening. It should be noted that the above results could not be obtained by the chemical analysis on the bulk flesh. Because fruit flesh is composed of a multiplicity and diversity of cells that have different metabolic levels, cellular level investigation of the cell degradation in fruit is of profound significance for deciphering the mechanisms underlying fruit softening during the postharvest stage. Moreover, it is interesting to correlate distribution changes of cell wall polysaccharides at the cell level with transcriptome profiles. There are some works published on peach transcriptome profiles [42,43,44,45]. Nevertheless, these works have mainly focused on melting peach. To date, there is no work on studying transcriptome profiles of nonmelting peach during the postharvest storage. In the future, the study on transcriptome profiles of nonmelting peach ”Zhonghuashoutao” during the softening process should be carried out to associate the results with the changes in cell wall polysaccharides of peach at single- and multiple-cell levels during the postharvest storage.

Although this work successfully used SRS to image the changes of the total cell wall polysaccharides in peach flesh at the massive-cell level, the current SRS system used in this work could not achieve the imaging of cellulose, hemicellulose, and pectin separately. This is because SRS hyperspectral imaging requires the simultaneous presence of both pump and Stokes laser beams, and detects the change of incident laser photon energy such as the loss of the former or the gain of the latter during the scattering process [46]. The parenchyma cells in fruit have large vacuoles and relatively low content of the compositions of cell walls and do not have secondary cell walls. When SRS was applied for analyzing fruit samples, the change of incident laser photon energy was very small, even less than one ten-thousandth of the original incident laser intensity [47], making it difficult to detect the signal of cellulose, hemicellulose, and pectin separately. In the future, more work should be put into the improvement of the SRS system to measure the weak signal of cellulose, hemicellulose, and pectin in the cell wall of parenchymal cells in fruit.

For the changes in the physiological attributes of peach fruit during the softening process, most previous works have studied melting peaches. This work first time studied the changes of cell wall polysaccharides in the softening process of ”Zhonghuashoutao” peach, which is a type of nonmelting type peach. Compared with the results of previous studies on melting peaches [48,49], Our results showed that the nonmelting peach also softened during the postharvest storage similar to melting peaches, but maintained relatively higher firmness (approximately 30 N) at the late stage. The firmness of peach fruit continuously decreased during the postharvest storage at both temperatures, and the low-temperature storage condition (0 °C) significantly delayed the decrease in firmness and prolonged the storage life of peach. According to the chemical analysis, the contents of three major cell wall polysaccharides, cellulose, hemicellulose, and total pectin, and two pectin fractions (CSP and DASP) were found to decrease during the postharvest storage, which could be the reason for the decrease in fruit firmness. Only the content of WSP fraction in nonmelting type peach increased during the postharvest storage, which indicated that mutual transformation of different types of pectins exists during the postharvest storage. In addition, this study shows that the storage time of nonmelting peaches at 20 °C could be over 30 days. On the contrary, for the melting peach, such as ”Hongli” and ”Ambra”, their storage life at 20 °C are usually less than a week [50,51].

Cell wall polysaccharides mainly include cellulose, hemicellulose, and pectin. In plant cell wall, hemicellulose is not a chemically well-defined compound but rather a family of polysaccharides, composed of different five-carbon (e.g., xylose and arabinose) and six-carbon (e.g., glucose, galactose, and mannose) monosaccharide units [16]. The hemicellulose of primary cell walls of dicots is mainly xyloglucan [23]. Many works have found that the xyloglucan, a generic name of linear polysaccharides consisting of (β1→4)-linked d-glucan, is the main hemicellulose of most vascular plants [1,3,4,5]. Glucuronoarabinoxylan and (Gluco) mannan also exist in primary cell walls of dicots, but their amounts are much less than xyloglucan [23]. Other hemicelluloses, such as glucuronoxylan, galactoglucomannan, and β-(1→3,1→4)-glucan are absent or minor in primary cell walls of dicots [23]. For the measurement of cell wall materials of this work, a hot alcohol-insoluble solid method was used to extract the cell wall polysaccharides. During the extraction, cytosolic components were removed after cell breakage, and the major cell wall polysaccharides in the residue were obtained. Then, the hemicellulose content could be reflected by the xyloglucan content. As xyloglucan has a backbone of glucose, the glucose content in hemicellulose content in the residue, determined by the anthrone method, was used to reflect the hemicellulose content. The anthrone method is commonly used to measure the hemicellulose content in fruit cell wall during the postharvest storage, including blueberries [52], pear [53], and sweet cherry [54]. It should be noted that although glucose was likely to be the main monosaccharide measured, other monosaccharides also contributed to the result of the anthrone method used in this work.

The research focus of this work is the evaluation of cell wall changes of peach fruit during storage. In addition to the softening during storage, mechanical damage and chilling injury can also cause changes in the cell wall of fruit. In particular, shelf-life treatment after cold storage is a method for evaluating the chilling tolerance of peach fruit after additional ripening at room temperature (i.e., 3 d shelf life). Nevertheless, although chilling injury could occur after shelf-life treatment, the study on shelf life is outside the scope of this manuscript. In this work, there was no incidence of chilling injury symptoms during the whole storage at 20 °C and 0 °C. In future works, CRM, FTIRM, and SRS could be used to further study the effects of mechanical damage and chilling injury on fruit texture from the perspective of cell wall changes. Moreover, the feasibility of using these techniques to obtain the distribution changes of intracellular compounds in fruit, such as flavonoids, essential oils, and carotenoids, could also be evaluated.

The results of the content distribution obtained by CRM, FTIRM, and SRS imaging were consistent with those of the chemical analysis. Moreover, CRM, FTIRM, and SRS imaging captured valuable distribution information, showing that the total polysaccharide compositions tended to degrade more rapidly in the intercellular layer and more slowly in the cell corner, and different parenchymal cells had different content and spatial change processes of cell wall polysaccharides. However, although histology-guided chemical analysis is usually time-consuming and laborious and can inevitably alter the native structure of the samples, it is still recommended that the compositions of homogenized bulk tissues should be measured in addition to the microscopic imaging of the cells to make the imaging results more credible.

## 5. Conclusions

In this study, to understand the softening process of a nonmelting peach named ”Zhonghuashoutao” at the cellular level, the degradation of cell wall compositions in flesh cells at the single-cell level and the massive-cell level during the softening process of the nonmelting peach after harvest was studied. The firmness results show that the nonmelting type peach softened during the postharvest storage but maintained relatively high firmness (approximately 30 N) at the late stage. The chemical analysis found that the contents of three major cell wall polysaccharides, cellulose, hemicellulose, and total pectin, and two pectin fractions (CSP and DASP) decreased during the postharvest storage. Based on the complement of CRM and FTIRM label-free hyperspectral molecular images, the content distribution of cellulose, hemicellulose, and pectin in the cell wall of peach flesh was visualized simultaneously during the softening process at the single-cell level, and the results show that the cell wall polysaccharides tended to be concentrated in the cell corner of parenchymal cells at the late stage. Furthermore, the overall trend of fruit softening was concluded based on SRS hyperspectral images of parenchymal cells at the massive-cell level. On the one hand, the SRS signal was also found higher in the cell corner than other parts of cell wall, which corroborated the results of CRM and FTIRM analysis. On the other hand, by analyzing the SRS images, it was further found that the degradation rates of cell walls and their time to start degradation were different and not all consistent with the overall trend of fruit softening. Moreover, the grayscale analysis based on SRS hyperspectral images further facilitated the objective and quantitative elucidation of the overall changes in cell wall polysaccharides of peach fruit during the postharvest storage. Because SRS has similar resolution but ultrafast imaging speed as compared with CRM (approximately three or four orders of magnitude), SRS is a promising method for high-throughput label-free cellular phenotypes measurement of physiological processes of fruit during the postharvest storage without ignoring the cell-to-cell difference. These findings show an important benefit to allow label-free spatial molecular hyperspectral imaging of fruit softening at the multiple cell levels as compared with standard chemical analysis, and the results show that differences in the content and spatial changes of cell wall polysaccharides existed among parenchymal cells of peach fruit during the softening process. The visualization of cell wall polysaccharides at both the single-cell and massive-cell levels enhances our understanding of the changes and differences of cells in the softening process of peach fruit intuitively.

## Supplementary Materials

The following are available online at https://www.mdpi.com/2073-4409/9/3/761/s1. Figure S1: Example of all Raman spectrum profiles in one CRM hyperspectral image of cell wall of peach before and after denoising (a), Example of one Raman spectra profile in (a) before and after baseline correction (b), Figure S2: The reference spectra of parenchymal cell wall of peach flesh, Figure S3: The Infrared spectroscopy of cell wall of peach fruit (a), and extracted hemicellulose and pectin (b), Figure S4: Details of SRS hyperspectral images of peach parenchyma tissue in the red box in Figure 4 at the initial stage (a and b), in the middle stage (c and d) and at the late stage (e and f) during peach softening at 0 °C and 20 °C. Since 0 d is the starting point of postharvest storage, images of 0 °C and 20 °C at 0 d were the same as they were obtained from the same samples., Figure S5: Changes in percentage of the number of pixels with all gray values (0–255) in SRS hyperspectral image of peach stored at 0 °C (a) and 20 °C (b) in the total number of pixels. (c) and (d) represent the red box in picture (a) and (b), which contain the changes in percentage of the number of pixels with all gray values (50–255).
