# Supplementary material for: Hybrid Label-Free Molecular Microscopies for Simultaneous Visualization of Changes in Cell Wall Polysaccharides of Peach at Single- and Multiple-Cell Levels during Postharvest Storage
Source: Cells. 2020 Mar 20;9(3):761. doi: 10.3390/cells9030761 (PMC7140658; doi:10.3390/cells9030761)
Supplement: Supplementary file 1 [file cells-09-00761-s001.zip › cells-703784-SI/Supplementary materials.docx]

**Hybrid label-free molecular microscopies for simultaneous visualization of changes in cell-wall polysaccharides of peach at single and multiple-cell levels during postharvest storage**

Weinan Huang^1a^, Yating Nie^1a^, Nan Zhu ^1^, Yifan Yang ^2^, Changqing Zhu^1^, Minbiao Ji^2^, Di Wu^1^*, Kunsong Chen^1^

^1^ College of Agriculture & Biotechnology/Zhejiang Provincial Key Laboratory of Horticultural Plant Integrative Biology/The State Agriculture Ministry Laboratory of Horticultural Plant Growth, Development and Quality Improvement, Zhejiang University, Zijingang Campus, Hangzhou 310058, P. R. China

^2^ State Key Laboratory of Surface Physics and Department of Physics, Human Phenome Institute, Multiscale Research Institute of Complex Systems, Key Laboratory of Micro and Nano Photonic Structures (Ministry of Education), Fudan University, Shanghai 200433, China

*Corresponding author. Tel: +86 571 88982226. E-mail: di_wu@zju.edu.cn, china.di.wu@gmail.com

^a^ Weinan Huang and Yating Nie contributed equally to this work.

**Supplementary figure:**


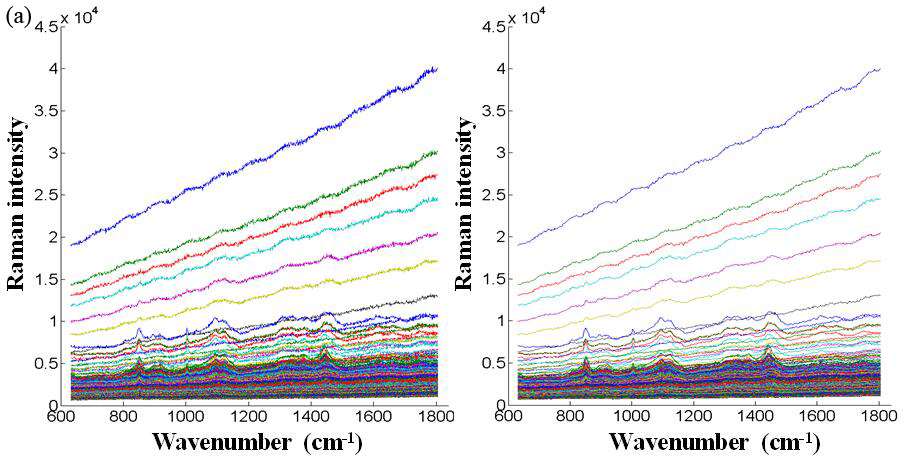


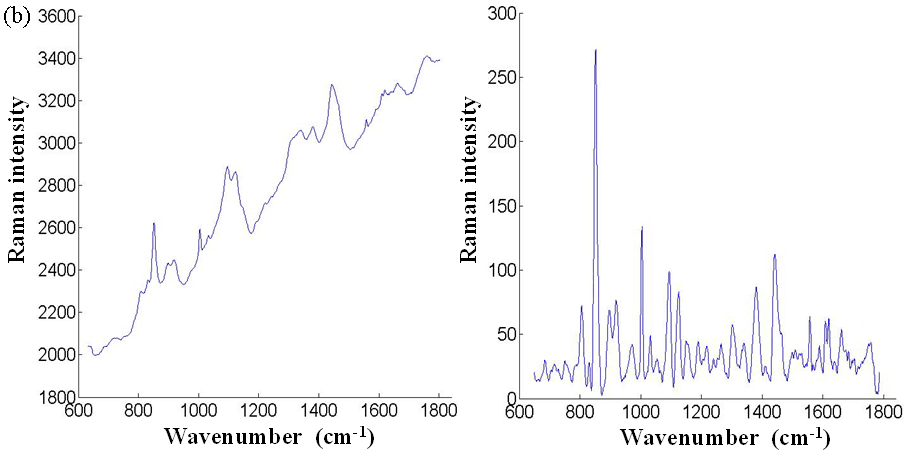
 Figure S1. Example of all Raman spectrum profiles in one CRM hyperspectral image of cell wall of peach before and after denoising (a), Example of one Raman spectra profile in (a) before and after baseline correction (b).


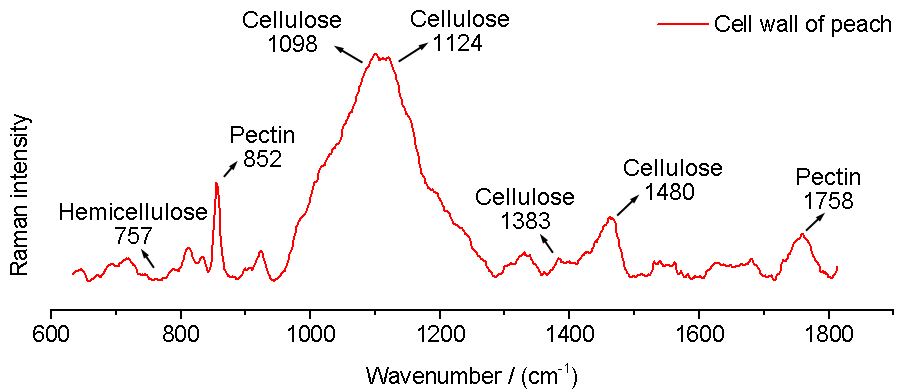


Figure S2. The reference spectra of parenchymal cell wall of peach flesh.


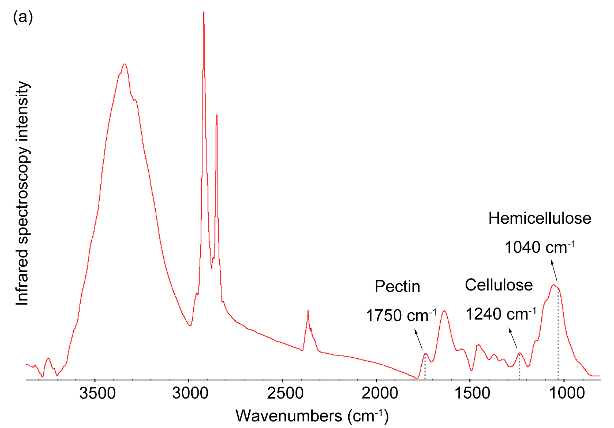

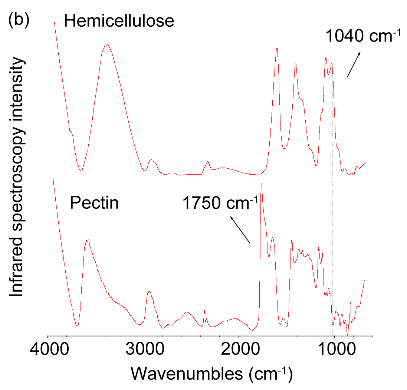


Figure S3. The Infrared spectroscopy of cell wall of peach fruit (a), and extracted hemicellulose and pectin (b).


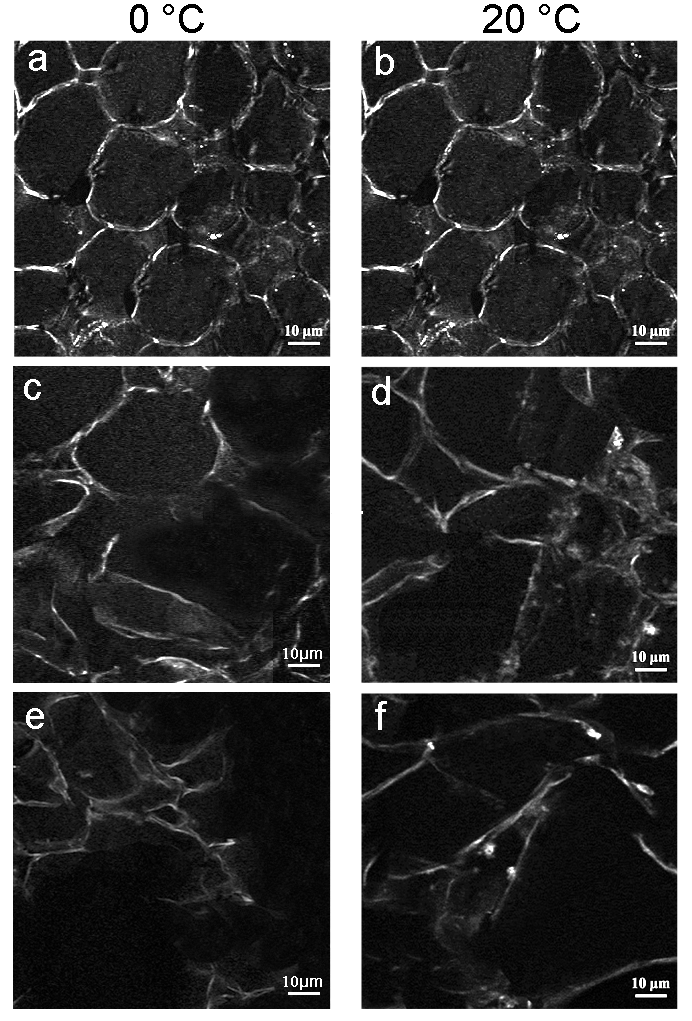


Figure S4. Details of SRS hyperspectral images of peach parenchyma tissue in the red box in Figure 4 at the initial stage (a and b), in the middle stage (c and d) and at the late stage (e and f) during peach softening at 0 °C and 20 °C. Since 0 d is the starting point of postharvest storage, images of 0 °C and 20 °C at 0 d were the same as they were obtained from the same samples.


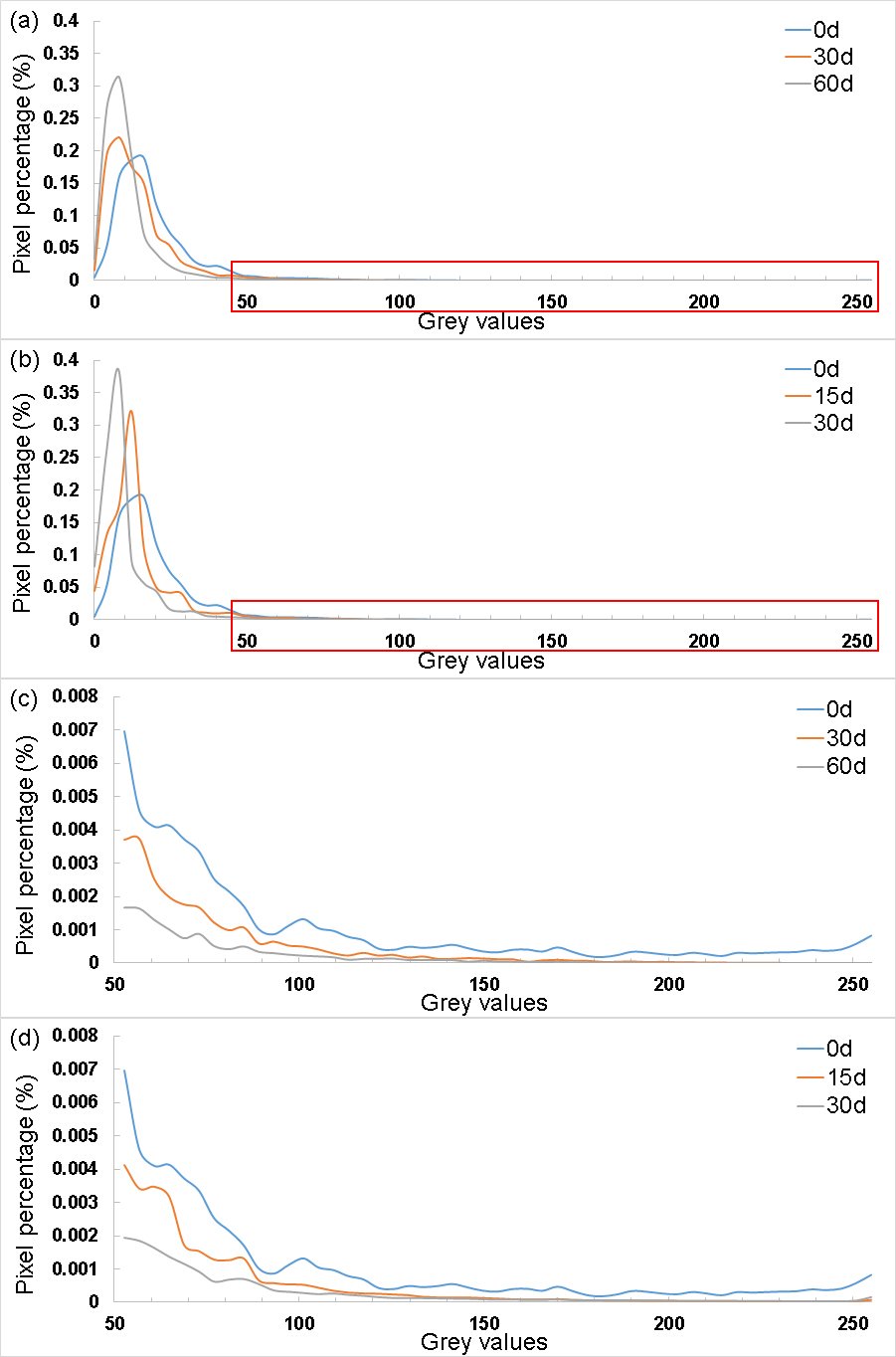


Figure S5. Changes in percentage of the number of pixels with all gray values (0-255) in SRS hyperspectral image of peach stored at 0 °C (a) and 20 °C (b) in the total number of pixels. (c) and (d) represent the red box in picture (a) and (b), which contain the changes in percentage of the number of pixels with all gray values (50-255).
