# Supplementary figures and images for: Hybrid Label-Free Molecular Microscopies for Simultaneous Visualization of Changes in Cell Wall Polysaccharides of Peach at Single- and Multiple-Cell Levels during Postharvest Storage
Source: Cells. 2020 Mar 20;9(3):761. doi: 10.3390/cells9030761 (PMC7140658; doi:10.3390/cells9030761)

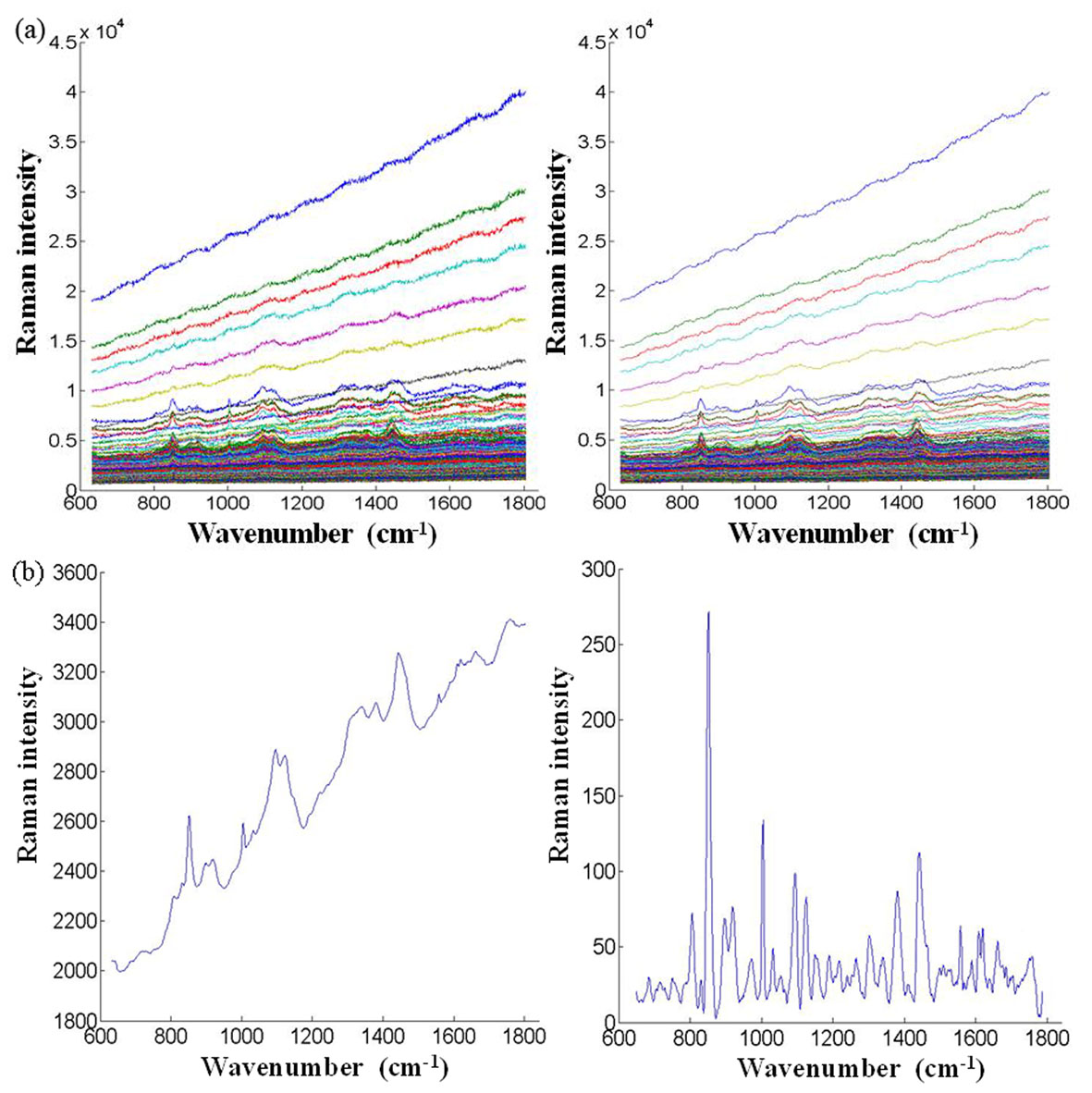

Supplement: Supplementary file 1 [file cells-09-00761-s001.zip › cells-703784-SI/Supplemental figure 1.jpg]

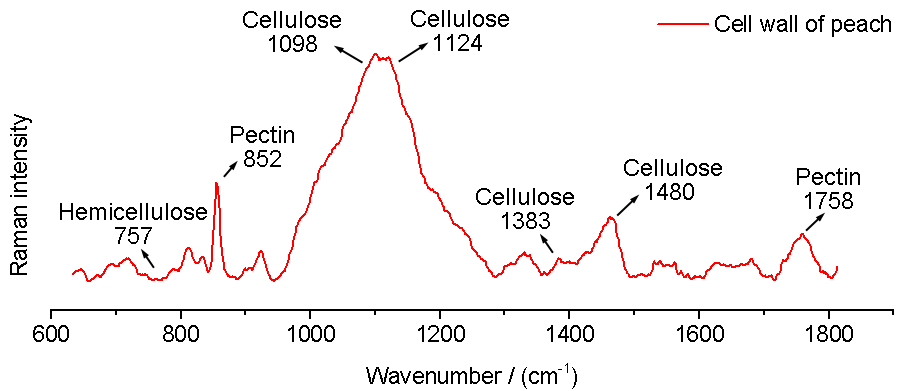

Supplement: Supplementary file 1 [file cells-09-00761-s001.zip › cells-703784-SI/Supplemental figure 2.tif]

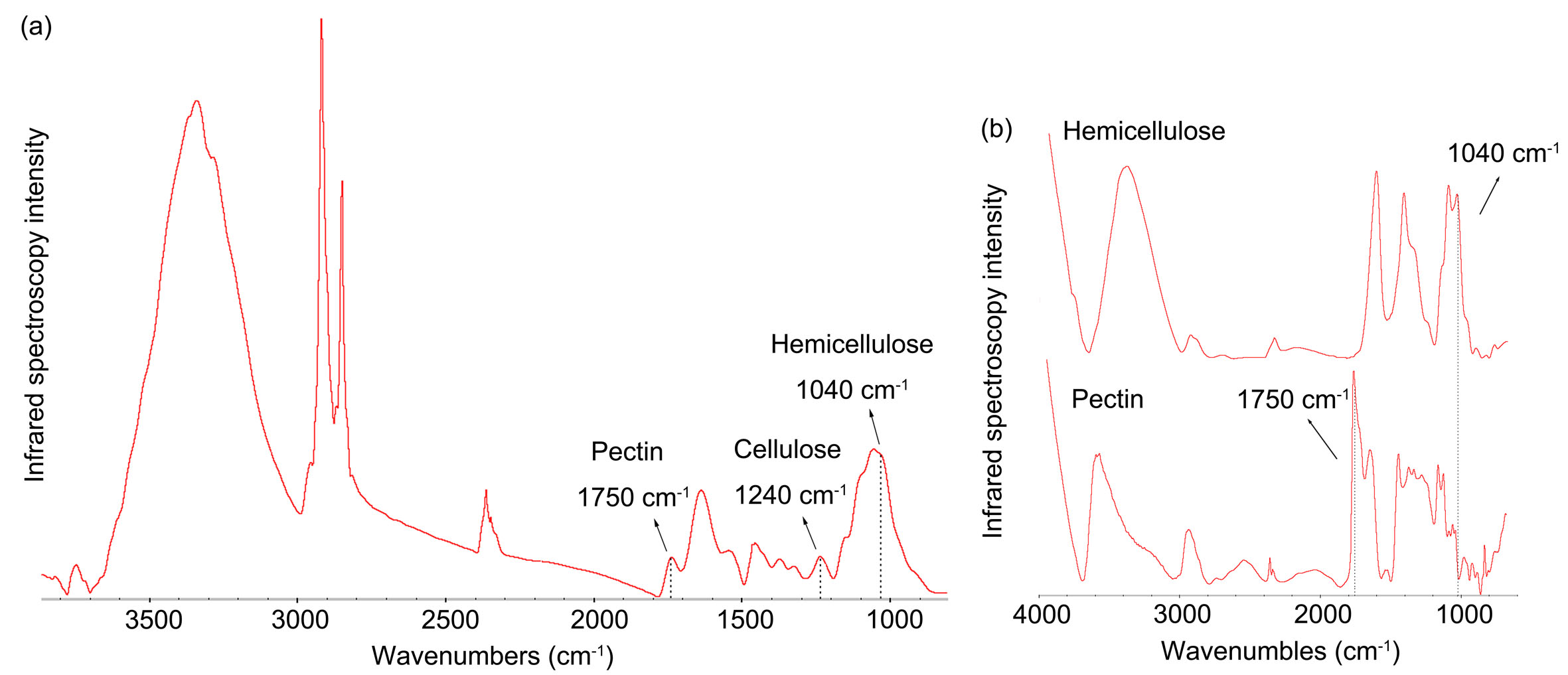

Supplement: Supplementary file 1 [file cells-09-00761-s001.zip › cells-703784-SI/Supplemental figure 3.jpg]

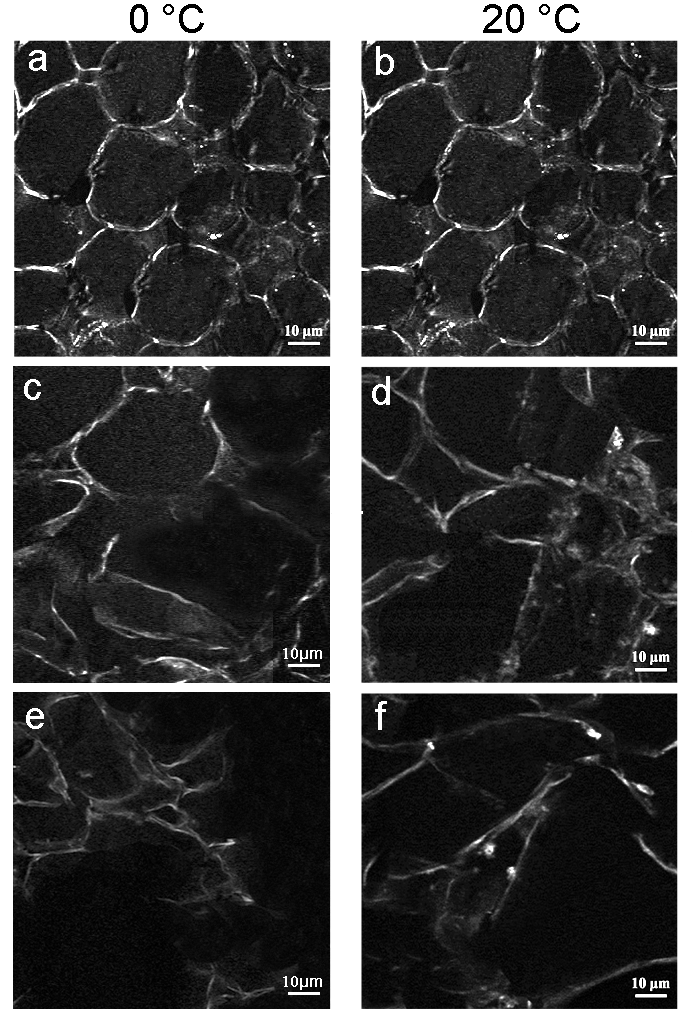

Supplement: Supplementary file 1 [file cells-09-00761-s001.zip › cells-703784-SI/Supplemental figure 4.tif]

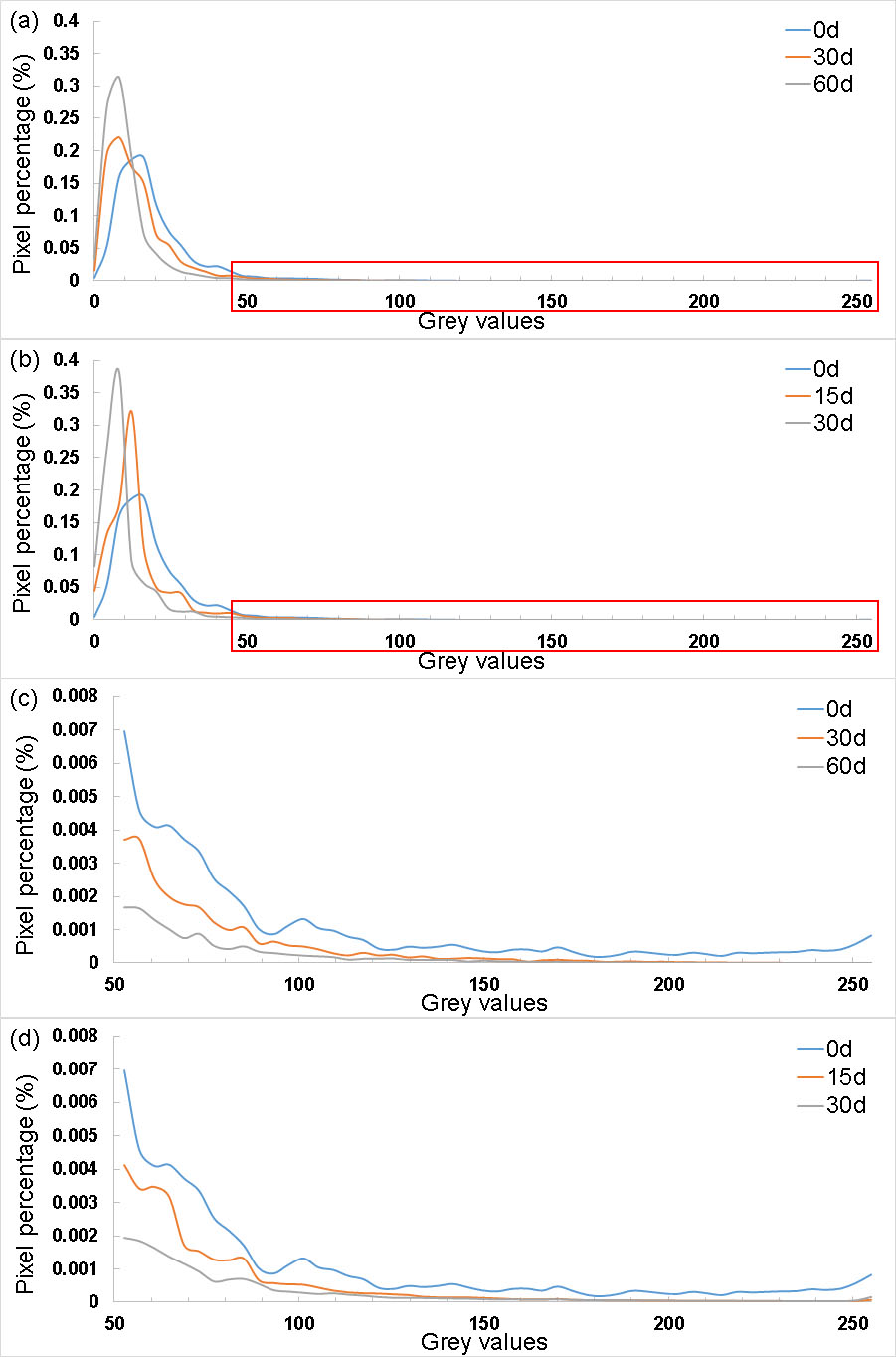

Supplement: Supplementary file 1 [file cells-09-00761-s001.zip › cells-703784-SI/Supplemental figure 5.jpg]
